# Ageing changes the proprioceptive contribution to balance control under different types of mastoid vibration: A cross‐sectional study

**DOI:** 10.1113/EP092548

**Published:** 2025-03-11

**Authors:** Haoyu Xie, Zhuo Wang, Chuhuai Wang, Jung Hung Chien

**Affiliations:** ^1^ Department of Rehabilitation Medicine the First Affiliated Hospital of Sun Yat‐Sen University Guangzhou Guangdong China; ^2^ Department of Health & Rehabilitation Science, College of Allied Health Professions University of Nebraska Medical Center Omaha Nebraska USA; ^3^ Department of Rehabilitation Medicine, West China Hospital Sichuan University Chengdu Sichuan China; ^4^ Independent Researcher Omaha Nebraska USA

**Keywords:** ageing, balance control, mastoid vibration, proprioception, sensory organization test, vestibular feedback

## Abstract

Ageing‐related sensory deteriorations are significantly associated with poor balance control among older individuals, resulting in a higher risk of falling in a dark environment. In particular, the proprioceptive system plays a critical role in maintaining balance. This study aimed to determine how ageing‐related sensory deteriorations contributed to balance control during standing under various sensory conflicts. Twenty healthy, active adults (10 young and 10 older) participated in this study. Balance control was quantified through two sensory organization test conditions (SOT‐1: unblindfolded standing; SOT‐2: blindfolded standing). Mastoid vibration (MV) was applied unilaterally (Uni) or bilaterally (Bi) to mastoid processes, for perturbing vestibular inputs. A total of six trials were assigned to each participant in a random order. Dependent variables included traveling route (TR), performance index (PI) and sample entropy (SaEn) in the anterior–posterior (AP) and medial–lateral (ML) directions. Our results showed that (1) compared to without MV, applying MV significantly increased TR_AP (Uni: *P *= 0.003; Bi: *P *< 0.001) and TR_ML (Uni: *P *= 0.009; Bi: *P *= 0.011) of all participants during blindfolded standing; (2) the application of Uni and Bi significantly increased PI_AP, PI_ML, SaEn_AP and SaEn_ML of young and older adults when standing in the SOT‐1 and SOT‐2 conditions (*P *< 0.05); and (3) older adults demonstrated significantly higher PI_AP, PI_ML and SaEn_ML than young adults in standing. This study indicated the potential risk of imbalance attributed to ageing‐related proprioceptive and vestibular deteriorations even in healthy older adults. Furthermore, unilateral MV had a stronger effect on disturbing ML balance control than bilateral MV.

## Abstract (highlights)

- **What is the central question of this study?**
  How do ageing‐related sensory deteriorations contribute to balance control of healthy older adults during standing in various sensory‐perturbed environments?
- **What is the main finding and its importance?**
  There is a potential risk of imbalance attributed to ageing‐related proprioceptive and vestibular deteriorations even in healthy older adults. Mastoid vibration was able to simulate a vestibular‐disrupted environment, thereby distrubing balance by increasing the magnitude and irregularity of center of gravity displacement.

## 1 INTRODUCTION

Falls have become the leading cause of accidental death among individuals aged 65 and older, with approximately 30% falling annually, potentially resulting in severe fall‐related sequelae and significant economic burdens (Ganz & Latham, 2020; Lamb et al., 2024). Despite various environmental and physiological causes of falls, a principal contributor is the ageing‐related decline in balance control (Muir et al., 2010). The maintenance of good balance in standing relies on sensory feedback collected from multiple systems (i.e., visual, proprioceptive and vestibular systems) (Lamb et al., 2024). These sensory inputs are sensitive to particular characteristics of self‐motion and environmental alterations, contributing to the detection of body sway to keep an upright posture (Chien et al., 2014). Then, after the processing and integration in the central nervous system (CNS), human bodies initiate corrective postural adjustments through appropriate locomotor commands (Ernst & Banks, 2002). Consequently, effective balance control necessitates collaborative sensorimotor systems and corresponding functions. However, the physiological changes associated with ageing result in the deterioration of the sensorimotor system in older adults. Specifically, ageing‐related proprioceptive deteriorations are widespread in the elderly, which causes serious postural instability and gait impairment while standing in a dark environment (Chatard et al., 2017; Doettl et al., 2021). Specifically, according to available evidence, approximately 50% of older adults (≥60 years) demonstrate varying degrees of sensory deficits, leading to a higher risk of falling and reduced quality of life (Ji & Zhai, 2018). With the growing worldwide ageing populations, research in the area of ageing and fall prevention continues to rise and becomes increasingly crucial. To explore the effect of ageing‐related sensory deterioration on balance control, multiple evaluations (e.g., clinical scales and questionnaires) are commonly used to indirectly identify or quantify various sensorimotor (balance) contributors, to develop targeted interventions to combat this decline in balance in clinical rehabilitation (Wagner et al., 2021).

The Sensory Organization Test (SOT) is a type of computerized dynamic posturography to quantify various sensory contributions and the adaptive mechanisms involved in postural control during standing, and has been widely used in the elderly and patients with impaired balance (e.g., Parkinson's disease and vestibulopathy) (Ford‐Smith et al., 1995; Nocera et al., 2009; Peterka & Black, 1990; Rossi‐Izquierdo et al., 2011). Through systematic manipulation of sensory inputs, SOT aims to perturb or block specific sensory systems and induces adaptive sensory recalibration processes. Among the six different SOT conditions, proprioceptive and visual inputs can be manipulated solely or jointly, allowing the assessment of an individual's ability to maintain balance under various sensory perturbations (Chien et al., 2014). The sensory reweighting hypothesis underlies the design of this paradigm. According to this hypothesis, each sensory channel is weighted in the CNS; then, the summed weighted variables create an appropriate response modulated by the reliability of the incoming afferents. Peterka (2002) suggested that when healthy adults stand on a firm surface with intact visual inputs (the SOT‐1, baseline condition), sensory contributions are distributed as follows: 70% from proprioception, 20% from vestibular feedback and 10% from vision. Nevertheless, when visual inputs are blocked through blindfolds (SOT‐2), sensory recalibration results in altered contributions to 65% proprioception, 35% vestibular feedback and 0% vision to maintain postural stability (Peterka, 2002). Based on these results, proprioceptive and vestibular systems seem to be the dominant sensory resources for achieving postural stability in daily standing, especially in a dark environment (Chien et al., 2014). However, although the proprioceptive contributions to balance control can be indirectly assessed through comparing the perturbation between SOT‐1 and SOT‐2, the ‘real’ contributions of vestibular or proprioceptive system still cannot be individually determined (Grace Gaerlan et al., 2012). This limitation emphasizes the need for understanding the highlighted proprioceptive contributions to standing balance, particularly through direct, simultaneous vestibular and visual perturbations.

Over the past decades, vestibular stimulation has been employed to directly manipulate vestibular inputs, leading to the identification of various vestibular disorders such as Meniere's disease, unilateral vestibulopathy and acute vestibular loss (Hong et al., 2007; Koo et al., 2011; Lee et al., 2015). Mastoid vibration (MV) is a vestibular stimulation method that applies vibrotactile stimuli to the mastoid processes for manipulating vestibular inputs (Lu et al., 2022). MV was first introduced by Lücke (1973) to detect abnormalities in peripheral vestibular function by applying 100 Hz vibration directly to the skull or mastoid processes to induce nystagmus. By measuring the characteristics of nystagmus, the various vestibular‐related disorders and dysfunctions can be diagnosed (Karlberg et al., 2003). Updated physiological evidence suggests that MV manipulates vestibular signals by inducing movement of the endolymphatic fluid and deflection of type I receptors at the striola – a specialized band of hair cells in the saccule and utricle (Curthoys, 2017). Therefore, MV is a feasible and cost‐effective method for applying vestibular stimulation and evaluating vestibular‐related balance control (Xie et al., 2024). According to our previously published studies, using MV (1) perturbs vestibular signals to identify alterations in postural control; and (2) induces different balance control strategies in various individuals (Lin et al., 2022; Lu et al., 2022). Specifically, MV significantly increases the area of centre of gravity (CoG) displacement in both young and older adults during standing; furthermore, applying bilateral MV results in greater CoG displacement than unilateral MV (Chien et al., 2016; Lin et al., 2022). Importantly, a paradigm that combines MV with posturography (i.e., SOT) can simulate an unpredictable vestibular‐disrupted environment, highlighting the different proprioceptive contributions for maintaining balance between healthy males and females (Zhang et al., 2024). However, knowledge regarding the use of MV combined with posturography to evaluate postural control related to ageing‐related deterioration in the proprioceptive system remains limited.

For interpreting SOT, a common technique is to measure the magnitude of CoG displacement in the anterior–posterior (AP) and medial–lateral (ML) directions (Cakmak et al., 2016). The total traveling route (TR) of CoG displacement in a given period is one of the most intuitive parameters used to quantify balance control in different SOT conditions, while a longer TR is interpreted as greater body sway in standing (Lemay et al., 2014). However, TR represents only the overall balance performance and cannot reveal instantaneous changes in CoG displacement through time series. Therefore, it is important to introduce advanced parameters for evaluating balance control. The performance index (PI) is calculated by the numerical integral of rectified CoG displacement signals scaled to a fraction of the maximal sway, which has been extensively employed to differentiate balance performance in different conditions or among different individuals (Black et al., 1989; Nashner et al., 1982). A greater PI reflects larger instantaneous body sway. In recent years, the entropy measure has been widely used to identify the complexity of physiological signals through time series (Blazkiewicz et al., 2021; Montesinos et al., 2018). Compared to conventional parameters of balance control calculated via means and standard deviations, the sample entropy (SaEn) method analysing CoG data through time series can assess the regularity/predictability of CoG displacement, providing another aspect of balance control to probe the underlying causes of systematic patterns over time (Zhang et al., 2024). The measurement of SaEn has the following advantages: (1) better data length independence, implying that the results of SaEn can ensure accuracy with a smaller error when applying to short datasets; and (2) advanced anti‐noise capacity and consistency (Montesinos et al., 2018; Richman et al., 2004). In general, more irregular motions associated with higher SaEn are commonly observed when adapting to the sensory‐conflicted environment, and vice versa for regular motions with lower SaEn (Chen et al., 2021). Our published studies have confirmed the feasibility of using SaEn to evaluate balance control among young and older groups to investigate the effect of sex differences or ageing (Lin et al., 2022; Zhang et al., 2024). On the basis of previous research, the present study plans to expand the application of MV to determine the role of proprioceptive feedback in balance control during standing under sensory perturbations using the above‐described measurements.

The purpose of this study was to determine the ageing‐related deteriorations in proprioceptive contributions to balance control through conventional SOT combined with different types of MV. We hypothesized that (1) even for healthy older adults with a moderate/high level of physical activity, the ageing‐related proprioceptive deterioration seemingly is inevitable, resulting in poorer balance control than young adults; and (2) regardless of standing in SOT‐1 or SOT‐2, applying MV would disturb the balance control but further highlight the proprioceptive contributions among healthy young and older adults.

## 2 METHODS

### 2.1 Participants

A total of 10 young (age: 22.20 ± 3.05 years; height: 170.80 ± 6.71 cm; weight: 66.80 ± 12.36 kg) and 10 older adults (age: 66.50 ± 4.55 years; height: 170.70 ± 6.17 cm; weight: 67.20 ± 12.14 kg) participated in this cross‐sectional study. The demographic characteristics (weight: *P *= 0.9727; height: *P *= 0.9426) and the sex distribution (6 males and 4 females, *P *= 1.0000) were homogeneous between two age groups. All participants recruited in this study were right‐handed. The advertising method via flyers posted in university campuses and local community centres was employed to recruit participants in this study. The inclusion criteria included that participants (1) were free from musculoskeletal disorders and had no history of joint injuries or replacements that may affect their balance; (2) had no history of visual, somatosensory or vestibular deficits; (3) had never experienced any type of vestibular stimulation; (4) passed the Dizziness Handicap Inventory (DHI) assessment (score = 0) to indicate the intact vestibular function; and (5) were defined as moderate‐ or high‐level of physical activity based on the assessment of International Physical Activity Questionnaire‐Short Form (IPAQ‐SF). The exclusion criteria were that participants had (1) a history of neurological disorders; (2) a history of vestibular diseases or relevant surgeries; (3) any significant pain in their trunks or lower extremities in the previous 3 months; and (4) a score above 0 in the DHI assessment to show potential deteriorations in the vestibular system (Zhang et al., 2024). The DHI included 25 questions with a total score of 100 to quantify the severity of dizziness and its impact on daily life (Jacobson & Newman, 1990). Based on previous studies, DHI had good test–retest reliability (intraclass correlation coefficient (ICC) = 0.91–0.97) and internal consistency (Cronbach's α = 0.87) (Emasithi et al., 2022; Koppelaar‐van Eijsden et al., 2022). The levels of physical activity among participants were assessed by IPAQ‐SF. The IPAQ‐SF was a self‐reported questionnaire including seven open‐ended questions about individuals’ levels of physical activity (Craig et al., 2003). Previous studies suggested that IPAQ‐SF exhibited moderate‐to‐good test–retest reliability (ICC = 0.52–0.81) and high validity (Pearson's *r *= 0.72) (Sember et al., 2020; Van Holle et al., 2015). In the IPAQ‐SF, the frequency and duration of low/moderate/vigorous activities were collected to calculate the total volume of physical activity in MET‐min/week. Subsequently, each participant was categorized into the low/moderate/high level of physical activity. In this study, the results of the IPAQ‐SF indicated that two age groups had similar levels of physical activity (*P *= 0.6212). Detailed demographic information about older and young participants is shown in Table 1.

**TABLE 1:** Demographic information about older and young participants in this study.

	Older adults (*n* = 10)	Young adults (*n* = 10)	*P*
**Age (years)**	66.50 ± 4.55	22.20 ± 3.05	<0.0001
**Sex (male/female)**	6/4	6/4	1.000
**Height (cm)**	170.70 ± 6.17	171.80 ± 6.71	0.973
**Weight (kg)**	67.20 ± 12.14	66.80 ± 12.36	0.943
**BMI (kg/m^2^)**	22.92 ± 2.93	22.75 ± 2.90	0.896
**Forms of exercise (no. of participants)**	Jogging (*n* = 4) Swimming (*n* = 3) Tennis (*n* = 2) Tai Chi (*n* = 1)	Running (*n* = 5) Swimming (*n* = 3) Basketball (*n* = 2)	
**IPAQ‐SF**			
Walking (MET‐min/week)	784.80 ± 49.46	1196.64 ± 86.66	<0.0001
Moderate activity (MET‐min/week)	450.80 ± 40.75	508.84 ± 92.21	0.085
Vigorous activity (MET‐min/week)	286.36 ± 70.13	372.80 ± 106.35	0.046
**Level of physical activity (low/moderate/high, no. of participants)**	0/8/2	0/6/4	0.621

*Note*: Data are shown as the mean ± SD. Abbreviations: BMI, body mass index; IPAQ‐SF, International Physical Activity Questionnaire‐Short Form; MET, metabolic equivalent of task.

This study was carried out in accordance with the *Declaration of Helsinki* with the approval of the University of Nebraska Medical Center Institutional Review Board (IRB no. 379‐17‐EP, Omaha, NE, USA). Informed consent was obtained from each participant at the day of the data collection. Participants were also allowed to withdraw from the study any time without providing a reason. The sample size of this study was calculated through G*Power (http://www.gpower.hhu.de/). By setting a power of 80% and a level of significance of 5% (two‐sided), recruiting 10 participants in each group could reach a large effect size *f *= 0.138 via the partial eta squared method for using the mixed rmANOVA.

### 2.2 Experimental set‐up

The Balance Master System 8.4 (NeuroCom International, Clackamas, OR, USA) was used to perform standing tasks through SOT. This system included a moveable visual surround and a support surface allowing participants to lean forward by a maximal range of 7° or backward by a maximal range of 5° in the AP direction. Two force plates (22.9 × 45.7 cm) were connected by a pin joint and used to record the CoG displacement at the sampling frequency of 100 Hz. SOT contained a total of six different conditions to quantitatively measure individual abilities to utilize visual, proprioceptive and vestibular feedback (Wrisley et al., 2007). In this study, the SOT‐1 (unblindfolded standing on a stationary platform, baseline condition) and SOT‐2 (blindfolded standing on a stationary platform) were used to identify the role of proprioceptive system during standing. A blindfold was used to block visual feedback in the SOT‐2 condition. According to available evidence, SOT‐2 is commonly performed to probe the contribution of vestibular and proprioceptive systems to standing balance in healthy individuals and older adults (Perucca et al., 2021; Wrisley et al., 2007).

**FIGURE 1:**
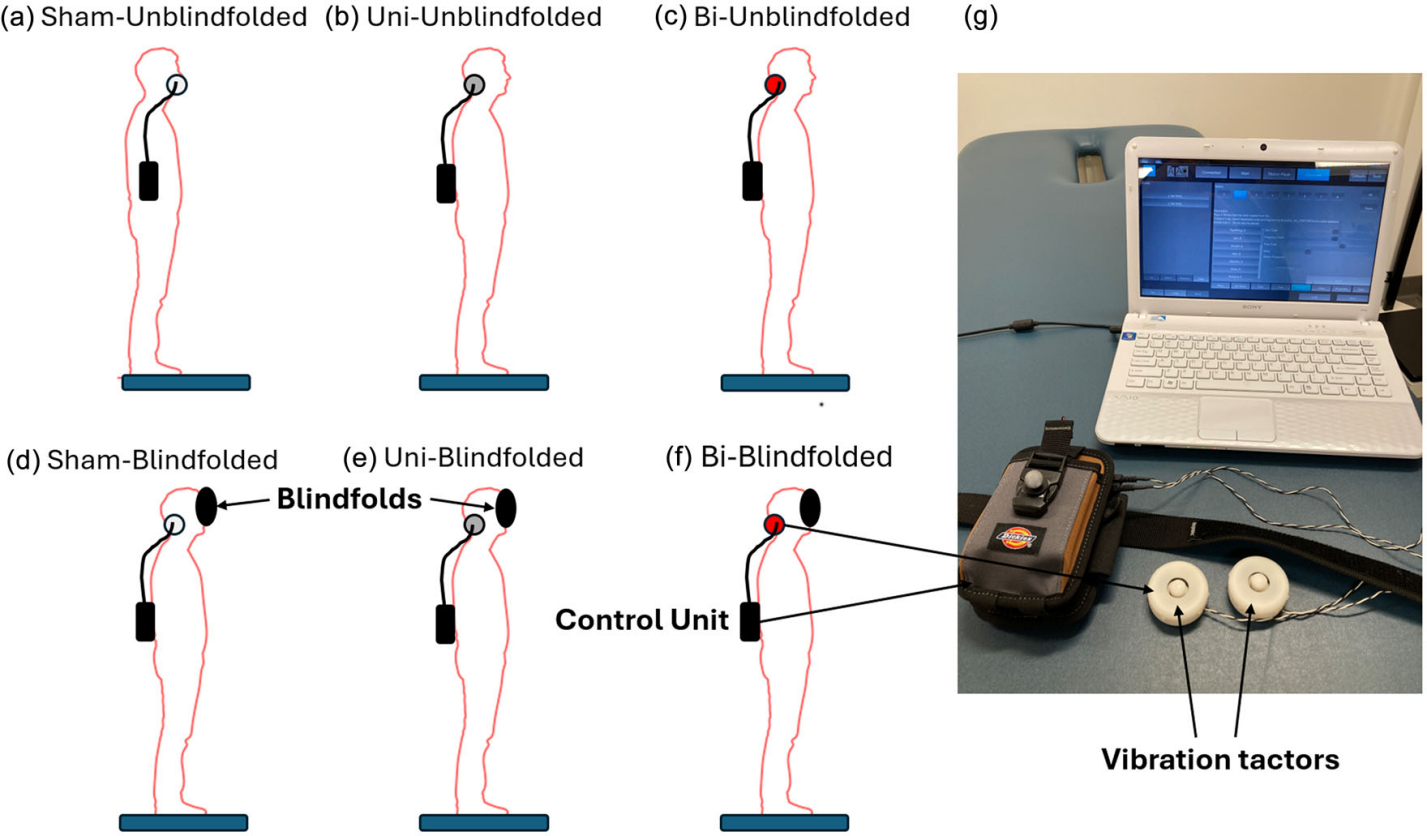
(a–f) The experimental protocol of different standing tasks used in this study; (g) the MV device for vestibular stimulation. Bi, bilateral MV; MV, mastoid vibration; Sham, without MV; Uni, unilateral MV.

Two electromechanical vibrotactile transducers (EMS^2^; Engineering Acoustics, Casselberry, FL, USA) were used to generate mechanical vibrotactile stimulation. Each tactor was 48.5 mm in diameter, 18.8 mm in height, and 24 g in weight (Figure 1). With the rise time of 25 ms, these tactors were designated to produce a maximal peak‐to‐peak displacement of 2 mm even when applied against mechanical impedances of human bodies. The TAction Creator software (Engineering Acoustics) was used to control MV set at the frequency of 100 Hz. Dumas et al. (2017) and Curthoys (2021) suggested that 100 Hz was the optimal frequency of MV to activate semicircular canal and otolith neurons and manipulate the vestibular feedback. Also, our previous publications had proven that MV at 100 Hz was able to induce a prominent body sway during standing or walking (Lu et al., 2022; Zhang et al., 2024). An impulsive mode of vibrotactile stimuli with an activation period of 0.5 s and a deactivation period of 0.5 s was used to reduce sensory saturation (Lu et al., 2022). The amplitude of MV was set to 130% of the individual vibrotactile thresholds, allowing participants to readily perceive the vibrotactile stimuli in this study (Severini & Delahunt, 2018). This amplitude of MV has been confirmed to act as an excitatory stimulus, successfully affecting vestibular perception and postural control (Kabbaligere et al., 2018). The protocol to determine the threshold described by Wuehr et al. (2016) was followed. Before the data collection, participants naturally stood with their head facing forward, eyes open and arms along the body. With the MV device placed well, individual threshold was established via a stepwise method: vibrotactile stimuli were delivered for a 10‐s period with the starting intensity of 0 dB. Then, the amplitude stepwise increased until participants perceived a minimal sensation at the tactor sites. The above‐described procedure was repeated three times and the average amplitude was defined as the final vibrotactile threshold. In this study, MV was applied unilaterally (Uni) or bilaterally (Bi) on each participant. For the sham condition (without MV), the MV device was properly worn by each participant but the amplitude was set as 0 dB to mitigate the placebo effect (Fonteneau et al., 2019). Uni was administered through one tactor placed on the right mastoid process to ensure consistency among participants; and Bi was implemented by placing two tactors on bilateral mastoid processes.

### 2.3 Experimental protocol

Before the commencement of data collection, participants were instructed to step each foot on one force plate without wearing shoes to adjust the foot placement. There were different markers on the force plates to help participants comfortably place their feet based on their heights. Participants’ bilateral ankle joints were required to be aligned with the horizontal line (*x*‐axis) running through the ML axis of the force plates. According to the method describe by Vanicek et al. (2013), participants positioned the medial malleolus of each foot over the horizontal line and placed the lateral side of calcaneus over the vertical line (*y*‐axis) marked by the T surface marking on each force plate. By achieving the foot placement based on this method, participants’ CoG should be located directly above the *x*‐ and *y*‐axis intercept, which acted as a reference point for the calculation of CoG displacement (Vanicek et al., 2013). Before data collection, participants were instructed to stand on the platform with eyes open for 10 s to define individual CoG_steady_state_. Then, they were required to lean their bodies forward/backward/lateral toward right/lateral toward left as far as possible (before taking a step or moving the feet). The above‐described four peaks were recorded to define CoG_max_.

**FIGURE 2:**
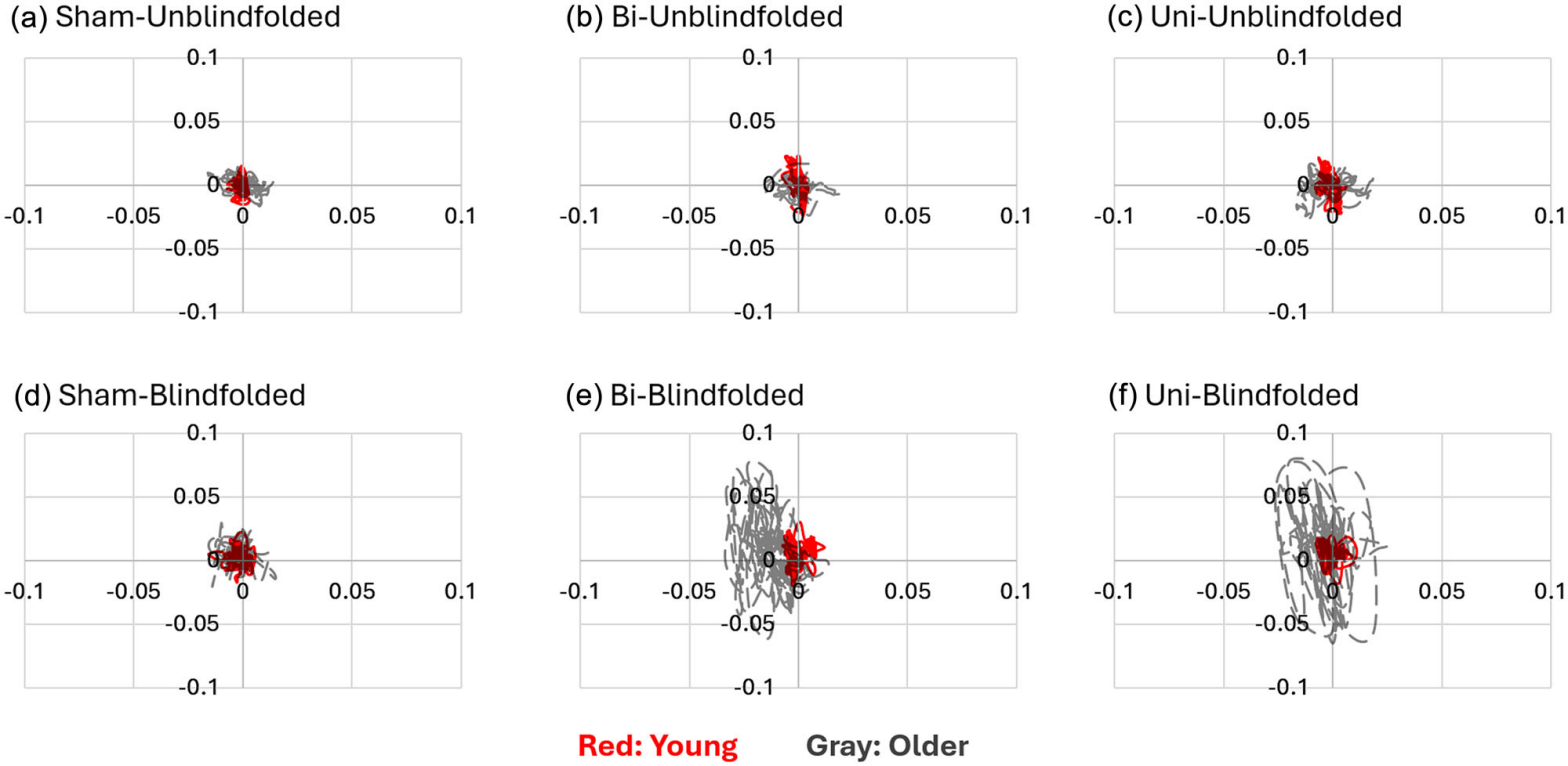
The trajectory diagrams of CoG displacement. The red and grey dashed lines represent healthy young and older participants, respectively. Bi, bilateral MV; CoG, centre of gravity; MV, mastoid vibration; Sham, without MV; Uni, unilateral MV.

In this study, a total of six standing trials (three types of MV × 2 SOT conditions) were assigned to each participant in a random sequence (Figure 1). For each trial, participants were required to stand upright with their hands on both sides, keep looking straight ahead, and refrain from moving their feet for 90 s. At the end of each trial, participants were asked whether they felt any uncomfortable sensations, such as nausea or dizziness. If so, the experiment would be terminated. A 1‐min mandatory rest was provided between two trials, and participants were allowed to take a longer rest if necessary (Lin et al., 2022). The whole experiment took approximately 1.5 h. All participants completed the data collection, and no incidents of falling or discomfort were observed. Figure 2 shows the protocol to present trajectories of CoG displacements in different trials from one young and one older participants.

### 2.4 Data analysis

All calculation scripts were written in MATLAB R2021a code (MathWorks Inc., Natick, MA, USA). In the present study, there were three components of measures to evaluate the balance control as follows: the total TR, PI and SaEn.

#### 2.4.1 Total TR of CoG displacement

The total TR was the sum of CoG displacement in the AP and ML directions within a time interval (Lemay et al., 2014). As the sampling frequency of force plates used in this study was 100 Hz, a time interval was 0.01 s. The total TR of CoG displacement was calculated in the AP and ML directions by following formula:

$$\mathrm{Total}\;\mathrm{Traveling}\;\mathrm{Route}\;\mathrm{of}\;\mathrm{CoG}=\sum_{t=0\;s}^{t=90\;s}\left|\mathrm{Co}G_{t+0.01\;s}-\mathrm{Co}G_{t}\right|$$

#### 2.4.2 PI of CoG displacement

The performance of balance control in standing was assessed using PI of CoG displacement. Conceptually similar to standard deviations, this metric was determined by the numerical integral of the rectified CoG displacement signals (removed from the steady‐state offset) and scaling the results as a percentage of maximum displacement during standing (Nashner et al., 1982). PI in the AP and ML direction was calculated via the following formula (Chien et al., 2014):

$$\mathrm{Performance}\;\mathrm{Index}\;=\sum_{t=0\;s}^{t=90\;s}\frac{\left|\mathrm{Co}G_{t}-\mathrm{Co}G_{\mathrm{steady}\_\mathrm{state}}\right|}{\mathrm{Co}G_{\max}-\mathrm{Co}G_{\mathrm{steady}\_\mathrm{state}}}$$

where CoG_steady_state_ was the averaged CoG coordinates in the AP and ML directions recorded before the data collection. The CoG_max_ in the AP direction was defined as the range between maximum lean forward and backward; the CoG_max_ in the ML direction was defined as the range between maximum lateral toward right and lateral toward left (Chien et al., 2014). PI ranged from 0 (stable balance control) to 100 (loss of balance), achieving the quantitative assessment of postural performance and sensory contributions in standing (Black et al., 1989; Zhang et al., 2024).

#### 2.4.3 SaEn of CoG displacement

The measurement of SaEn was used to evaluate the regularity within CoG displacement in time series collected under different conditions or in different groups. SaEn was defined as the negative natural logarithm for the conditional properties that a series of data pointed a certain distance apart, *m*, would repeat itself at *m* *+* *1*. Moreover, SaEn took the logarithm of the sum of conditional probabilities as SaEn (*m*, *γ*, *τ*, *N*), where *m* is data point length, *γ* is tolerance, *τ* is the time delay, and *N* is a time‐series data set of length. Given that $N=\{x_{1},x_{2},x_{3},\text{…}\text{…}x_{N}\}\;$ with a constant time interval, the template vector of length *m* was defined as $x_{m}\;\left(i\right)=\left\{x_{i},x_{i+1},x_{i+2},\text{…}\text{…}x_{i+m-1}\right\}$ and the distance function as $d[x_{m}\left(i\right),x_{m}\left(j\right)]$. Then, SaEn could be calculated by the following formula:

$$\mathrm{Sample}\;\mathrm{Entropy}=-\ln\frac{d\left[X_{m+1}\left(i\right),X_{m+1}\left(j\right)\right]}{d\left[X_{m}\left(i\right),X_{m}\left(j\right)\right]}$$

where both $d[x_{m}\left(i\right),x_{m}\left(j\right)]$ and $d[x_{m+1}\left(i\right),x_{m+1}\left(j\right)]$ were smaller than *γ*. *γ* = 0.2 and *m *= 3 were set in this study, following Zhang et al. (2024). In their study, applying *γ* = 0.2 and *m *= 3 could identify the subtle differences in CoG displacement during standing between healthy young males and females. Also, the present study followed Lin et al. (2022) to apply *τ* = 5 to CoG data in the AP and ML directions. After the adjustment of CoG data from 100 to 20 Hz via such a time delay (*τ* = 5), *N* was defined as a data set with 1800 data points (20 Hz × 90 s) (Lin et al., 2022; Montesinos et al., 2018).

### 2.5 Statistical analysis

There were six dependent variables in this study including total TR, PI, and SaEn of CoG displacement in the AP and ML directions. Each dependent variable was tested for normality via the Shapiro–Wilk normality test. When the data were normally distributed, a three‐way mixed rmANOVA with one between‐subject factor (two age groups) and two within‐subject factors (2 SOT conditions × 3 MV) was utilized to investigate the interaction on the above‐described dependent variables. When there was a significant interaction, *post hoc* comparisons were performed via the Bonferroni correction with adherence to guideline from IBM SPSS Statistics (IBM Corp., Armonk, NY, USA) (https://www.ibm.com/support/pages/calculation‐bonferroni‐adjusted‐p‐values). It should be noted that there were 12 pairwise comparisons in this study; therefore, the original (unadjusted) *P*‐values were multiplied by 12 to calculate the adjusted *P*‐values for *post hoc* comparisons. Only when the adjusted *P*‐values were less than 0.05 was the difference considered significant. In the event of the non‐normally distributed data, a non‐parametric longitudinal data model from Brunner et al. (2002) was used for investigating the within‐subject effect (2 SOT conditions × 3 MV) and the between‐subject effect (two age groups). If a significant interaction existed, the Wilcoxon signed‐rank test was applied for *post hoc* comparisons for the effect of different SOT conditions in each age group. To compare the effect of ageing in each SOT condition with/without different MV, the Mann–Whitney *U*‐test was performed. Additionally, an independent Student's *t*‐test was used to compare the weight and height between young and older adults. We also performed the partial eta squared method to understand the effect size based on Cohen's guidelines, in which 0.138 was considered a large effect size, 0.059 a moderate effect size and 0.01 a small effect size (Cohen, 1988). Statistical analysis was completed in SPSS 20.0. The significance level was set at 0.05.

In this study, the α values of the Shapiro–Wilk normality test were 0.124 for TR_AP, 0.235 for TR_ML, 0.089 for PI_AP, 0.078 for PI_ML, 0.432 for SaEn_AP and 0.330 for SaEn_ML, indicating that all data were normally distributed in this study. Therefore, a three‐way mixed rmANOVA was applied to investigate the interaction on above‐described dependent variables. Partial eta squared values were 0.279 for TR_AP, 0.201 for TR_ML, 0.187 for PI_AP, 0.249 for PI_ML, 0.464 for SaEn_AP and 0.733 for SaEn_ML, indicating large effect sizes based on Cohen (1988).

## 3 RESULTS

### 3.1 Total TR of CoG displacement

**FIGURE 3:**
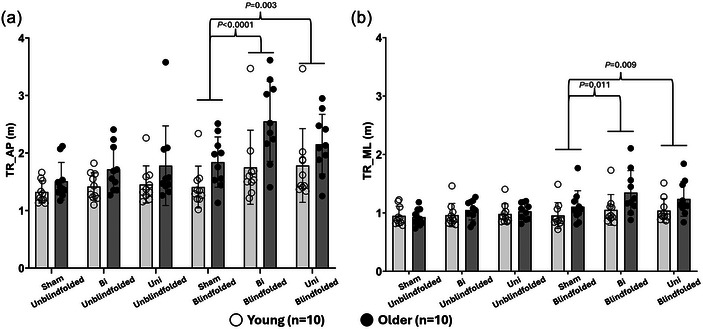
Total TR of centre of gravity in the AP and ML directions. AP, anterior–posterior; Bi, bilateral MV; ML, medial–lateral; MV, mastoid vibration; O, older participants; Sham, without MV; TR, traveling route; Uni, unilateral MV; Y, young participants.

A significant two‐way interaction (MV×SOT) was observed only in the total TR_AP (*F*_2,36 _= 6.966, *P *= 0.003) and TR_ML (*F*_2,36 _= 4.536, *P *= 0.017). No other significant two‐way interactions (TR_AP: Age×MV: *P = *0.165; Age×SOT: *P *= 0.192; TR_ML: Age×MV: *P = *0.230; Age×SOT: *P *= 0.375) or main effect of age groups (TR_AP: *P *= 0.069; TR_ML: *P *= 0.137) were found. Based on *post hoc* comparisons, applying Uni and Bi significantly increased TR_AP (Uni: *P *= 0.003; Bi: *P *< 0.0001) and TR_ML (Uni: *P *= 0.009; Bi: *P *= 0.011) of participants during blindfolded standing (SOT‐2). Additionally, for all participants, blindfolded standing significantly increased TR_AP when without MV (*P *= 0.039) or applying Bi (*P *= 0.001) and TR_ML when applying Bi (*P *= 0.048) in comparison to unblindfolded standing. More details are shown in Figure 3 and Tables 2, 3.

**TABLE 2:** Summary of total TR, PI, and SaEn data in the AP and ML directions for young and older participants.

	Sham‐unblindfolded		Bi‐unblindfolded		Uni‐unblindfolded		Sham‐blindfolded		Bi‐blindfolded		Uni‐blindfolded	
	Young	Older	Young	Older	Young	Older	Young	Older	Young	Older	Young	Older
**TR_AP (m)**	1.32 ± 0.19	1.51 ± 0.33	1.42 ± 0.24	1.72 ± 0.40	1.45 ± 0.32	1.78 ± 0.69	1.41 ± 0.36	1.84 ± 0.44	1.75 ± 0.64	2.55 ± 0.70	1.78 ± 0.64	2.15 ± 0.52
**TR_ML (m)**	0.95 ± 0.16	0.93 ± 0.14	0.96 ± 0.20	1.05 ± 0.17	0.98 ± 0.17	1.03 ± 0.14	0.96 ± 0.22	1.11 ± 0.27	1.05 ± 0.26	1.35 ± 0.38	1.04 ± 0.21	1.24 ± 0.30
**PI_AP**	7.23 ± 1.45	16.22 ± 2.08	11.76 ± 3.38	24.55 ± 3.46	10.84 ± 3.19	20.61 ± 3.40	10.00 ± 2.43	24.04 ± 3.92	16.02 ± 2.70	35.62 ± 2.60	12.94 ± 2.82	28.33 ± 3.00
**PI_ML**	5.42 ± 2.13	17.04 ± 4.05	8.46 ± 3.52	22.73 ± 4.27	9.41 ± 3.85	26.56 ± 4.75	9.20 ± 3.46	20.12 ± 4.37	15.19 ± 5.48	28.26 ± 4.79	17.11 ± 6.12	34.37 ± 3.05
**SaEn_AP**	0.120 ± 0.032	0.206 ± 0.051	0.153 ± 0.039	0.157 ± 0.041	0.176 ± 0.037	0.172 ± 0.057	0.143 ± 0.031	0.174 ± 0.047	0.095 ± 0.014	0.124 ± 0.029	0.128 ± 0.024	0.136 ± 0.033
**SaEn_ML**	0.110 ± 0.025	0.093 ± 0.031	0.141 ± 0.042	0.138 ± 0.037	0.183 ± 0.018	0.145 ± 0.035	0.209 ± 0.031	0.130 ± 0.048	0.107 ± 0.024	0.200 ± 0.043	0.135 ± 0.036	0.222 ± 0.020

*Note*: Data are shown as the mean ± SD. Abbreviations: AP, anterior‐posterior; Bi, bilateral mastoid vibration; ML, medial–lateral; PI, performance index; SaEn, sample entropy; Sham, without mastoid vibration; TR, traveling route; Uni, unilateral mastoid vibration.

**TABLE 3:** The statistical results of three‐way mixed rmANOVA using between‐subject factor (age) and within‐subject factors (MV; SOT conditions) for total route (TR), PI and SaEn in the AP and ML directions.

Dependent variables	Effect		df	*F* value	*P*‐value
**TR_AP (m)**	Three‐way interaction	Age×MV×SOT	2, 36	1.063	0.356
	Two‐way interaction	Age×MV	2, 36	1.895	0.165
		MV×SOT	2, 36	6.966	0.003^*^
		Age×SOT	1, 36	1.768	0.192
	Main effect	Age	1, 36	3.513	0.069
		MV	2, 36	5.538	0.008^*^
		SOT	1, 36	5.554	0.024^*^
**TR_ML (m)**	Three‐way interaction	Age×MV×SOT	2, 36	0.730	0.489
	Two‐way interaction	Age×MV	2, 36	1.531	0.230
		MV×SOT	2, 36	4.536	0.017^*^
		Age×SOT	1, 36	0.807	0.375
	Main effect	Age	1, 36	2.313	0.137
		MV	2, 36	5.248	0.010^*^
		SOT	1, 36	4.446	0.042^*^
**PI_AP**	Three‐way interaction	Age×MV×SOT	2, 36	4.147	0.024^*^
	Two‐way interaction	Age×MV	2, 36	2.801	0.074
		MV×SOT	2, 36	3.756	0.033
		Age×SOT	1, 36	5.243	0.028^*^
	Main effect	Age	1, 36	44.889	<0.0001^*^
		MV	2, 36	5.917	0.006
		SOT	1, 36	4.003	0.053
**PI_ML**	Three‐way interaction	Age×MV×SOT	2, 36	5.956	0.006^*^
	Two‐way interaction	Age×MV	2, 36	7.417	0.002^*^
		MV×SOT	2, 36	2.589	0.089
		Age×SOT	1, 36	4.746	0.036^*^
	Main effect	Age	1, 36	73.264	<0.0001^*^
		MV	2, 36	4.911	0.013^*^
		SOT	1, 36	0.315	0.578
**SaEn_AP**	Three‐way interaction	Age×MV×SOT	2, 36	15.597	0.0001^*^
	Two‐way interaction	Age×MV	2, 36	4.573	0.017^*^
		MV×SOT	2, 36	1.296	0.286
		Age×SOT	1, 36	9.458	0.004^*^
	Main effect	Age	1, 36	13.389	0.0008^*^
		MV	2, 36	7.417	0.002^*^
		SOT	1, 36	1.674	0.204
**SaEn_ML**	Three‐way interaction	Age×MV×SOT	2, 36	49.462	<0.0001^*^
	Two‐way interaction	Age×MV	2, 36	6.160	0.005^*^
		MV×SOT	2, 36	2.756	0.077
		Age×SOT	1, 36	8.944	0.005^*^
	Main effect	Age	1, 36	5.174	0.029^*^
		MV	2, 36	6.854	0.003^*^
		SOT	1, 36	1.887	0.178

Abbreviations: AP, anterior–posterior; df, degree of freedom; ML, medial–lateral; MV, mastoid vibration; PI, performance index; SaEn, sample entropy; SOT, sensory organization test. *Statistical significance.

### 3.2 PI of CoG displacement

**FIGURE 4:**
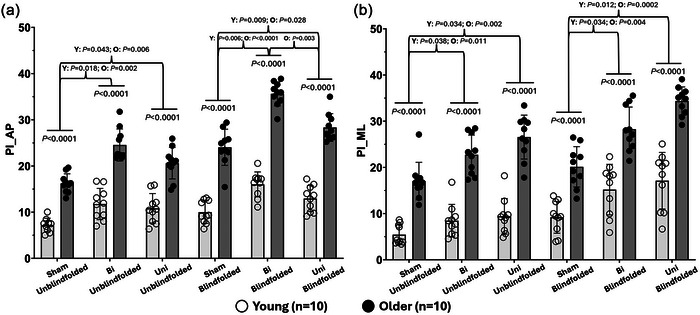
PI of centre of gravity in the AP and ML directions. AP, anterior‐posterior; Bi; bilateral MV; ML, medial‐lateral; MV, mastoid vibration; O; older participants; PI, performance index; Sham; without MV; Uni; unilateral MV; Y; young participants.

A significant three‐way interaction (MV×SOT×group) was observed in PI_AP (*F*_2,36 _= 4.147, *P *= 0.024) and PI_ML (*F*_2,36 _= 5.956, *P *= 0.006). *Post hoc* comparisons indicated that (1) older adults demonstrated significantly higher PI_AP and PI_ML than young adults in all conditions (*P *< 0.0001), suggesting a poorer balance control while standing with and without MV; (2) during unblindfolded standing, applying Uni and Bi significantly increased PI_AP (young: Uni: *P *= 0.043; Bi: *P *= 0.018; older: Uni: *P *= 0.006; Bi: *P *= 0.002) and PI_ML (young: Uni: *P *= 0.034; Bi: *P *= 0.038; older: Uni: *P *= 0.002; Bi: *P *= 0.011) compared with without MV in two groups; (3) during blindfolded standing, the application of Uni and Bi demonstrated significantly greater PI_AP (young: Uni: *P *= 0.009; Bi: *P *= 0.0006; older: Uni: *P *= 0.028; Bi: *P *< 0.0001) and PI_ML (young: Uni: *P *= 0.012; Bi: *P *= 0.034; older: Uni: *P *= 0.0002; Bi: *P *= 0.004) than without MV in two groups; and (4) in the SOT‐2 condition, Bi also significantly increased PI_AP compared with Uni in young (*P *= 0.019) and older adults (*P *< 0.0001). Additionally, in comparison to unblindfolded standing, blindfolded standing significantly increased PI_AP of older adults (Sham: *P *= 0.004; Uni: *P *= 0.0005; Bi: *P *< 0.0001) and PI_ML of young adults (Sham: *P *= 0.032; Uni: *P *= 0.007; Bi: *P *= 0.005) with and without the application of MV. More details are presented in Figure 4 and Tables 2, 3.

### 3.3 SaEn of CoG displacement

**FIGURE 5:**
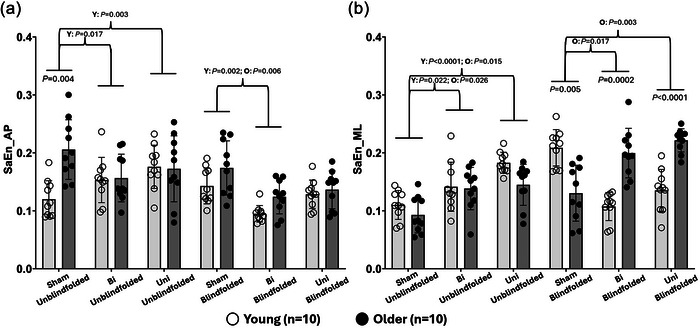
SaEn of centre of gravity in the AP and ML directions. AP, anterior‐posterior; Bi, bilateral MV; ML, medial‐lateral; MV, mastoid vibration; O, older participants; SaEn, sample entropy; Sham, without MV; Uni, unilateral MV; Y, young participants.

Significant three‐way interactions (MV×SOT×group) were observed in SaEn_AP (*F*_2,36 _= 15.597, *P *= 0.001) and SaEn_ML (*F*_2,36 _= 49.462, *P *< 0.0001). *Post hoc* comparisons showed that (1) compared to young adults, older adults demonstrated significantly greater SaEn_AP (*P *= 0.004) in SOT‐1 but lower SaEn_ML (*P *= 0.005) in SOT‐2 without applying MV; (2) while standing in SOT‐2 with the application of Uni and Bi, SaEn_ML of older adults was significantly higher than that of young adults (Uni: *P *= 0.0002; Bi: *P *< 0.0001); (3) during unblindfolded standing, applying Uni and Bi significantly increased SaEn_AP of young adults (Uni: *P *= 0.003; Bi: *P *= 0.017) and SaEn_ML of two age groups (young: Uni: *P *< 0.0001; Bi: *P *= 0.022; older: Uni: *P *= 0.015; Bi: *P *= 0.026) compared with without MV; (4) in SOT‐2, applying Bi significantly decreased SaEn_AP compared with without MV among young (*P *= 0.002) and older adults (*P *= 0.006); and (5) applying MV significantly increased SaEn_ML of older adults when standing blindfolded in comparison to without MV (Uni: *P *= 0.003; Bi: *P *= 0.017). Moreover, compared to unblindfolded standing, blindfolded standing significantly increased SaEn_ML of older adults (Uni: *P *< 0.0001; Bi: *P *= 0.035) with the application of MV. More details are presented in Figure 5 and Tables 2, 3.

## 4 DISCUSSION

This study investigated the effect of ageing‐related proprioceptive deteriorations to balance control through SOT‐1 and SOT‐2 conditions under various types of MV. The present results mostly confirmed our hypotheses that (1) in comparison to young adults, healthy older adults in moderate/high level of physical activity demonstrated altered proprioceptive contributions and poorer balance, as evidenced by significantly greater PI and SaEn in the AP and ML directions; and (2) the application of MV significantly disturbed the balance control of both groups by increasing PI and SaEn regardless of the SOT conditions in which participants stood; as a trade‐off, participants further relied on the proprioceptive feedback to maintain standing balance.

### 4.1 The ageing‐related proprioceptive deterioration was inevitable even though older adults were in moderate/high level of physical activity

According to available evidence, maintaining a moderate/high level of physical activity has multiple benefits for adults aged 65 and over, including improving general health and preventing various chronic diseases (Bherer et al., 2013). Additionally, it has been confirmed as a promising non‐pharmaceutical intervention to prevent ageing‐related sensorimotor decline, to enhance balance, and to reduce the risk of falling (Patti et al., 2021). Boulares et al. (2023) applied a 2‐month multi‐component physical activity programme to older individuals with mild cognitive impairment and suggested that this programme targeted the ageing‐related deterioration of proprioception and strength, thereby improving sensorimotor function and balance. Consequently, older adults engaged in more physical activities were expected to be less affected by the ageing‐related sensorimotor decline (Papp et al., 2022). However, the present study revealed that even among healthy older adults in a moderate/high level of physical activity, ageing still deteriorated the proprioceptive system, regardless of whether sensory perturbations were applied. Since the proprioceptive feedback played an important role in the maintenance of balance control, the ageing‐related proprioceptive deterioration among older adults resulted in reduced proprioceptive inputs and/or sensory integration capability, which disturbed balance control, as evidenced by significantly greater PI than young adults in all conditions (Chatard et al., 2017). Goble et al. (2009) supported this hypothesis by verifying that a rapid decline in proprioceptive sensibility occurred among individuals aged 60 and above, whose consequences were impairments of balance control, particularly during standing and walking. Moreover, Vaughan et al. (2017) substantiated in mice that ageing‐related physiological changes preferentially led proprioceptive sensory neurons to degenerate, causing deficits in sensorimotor function and balance. It should be noted that such ageing‐related proprioceptive deteriorations were observed to precede the ageing of muscle fibres, suggesting that the decline in proprioception among the elderly already demonstrated an impact on motor function and balance prior to the degeneration of the musculoskeletal system (e.g., sarcopenia) (Henry & Baudry, 2019; Vaughan et al., 2017). Therefore, in conjunction with this study, the simultaneous application of vestibular and visual perturbations during standing further highlighted the effect of the deteriorated proprioceptive system and/or sensory integration capabilities on balance control in older adults (Seidler et al., 2010; Wang, Li, et al., 2024). Under these circumstances, they had to rely more on the reduced proprioceptive feedback to maintain balance; as a result, significantly increased body sway in standing was observed. Additionally, older adults also performed more unpredictable movement patterns via an exploratory approach to maintain balance than young adults, as evidenced by higher SaEn while applying MV (Chien et al., 2017, 2016). Another potential explanation was that ageing‐related vestibular deteriorations in older adults led to poorer vestibular perception with the application of MV. Consequently, balance control among older adults exhibited a greater susceptibility to disturbance from MV. It could be concluded that even for healthy older adults in a high level of physical activity, the ageing‐related proprioceptive and vestibular deteriorations were inevitable. Moreover, significant differences in balance control between two age groups underscored the importance of developing age‐specific reference standards for clinical balance tests to differentiate between healthy older adults and those with impaired balance (Åhman et al., 2021). Most normative data for clinical balance tests were established based on young adults, neglecting the impact of ageing‐related sensory deteriorations, potentially leading to false positive results among healthy older individuals (Rosa et al., 2019). Therefore, identifying postural changes associated with ageing offered insights into the role of ageing‐related sensory deteriorations on balance control during locomotion (Michalska et al., 2021; Yang et al., 2024). Our results could serve as fundamental references for future comparisons between pathological and healthy elderly populations, as well as the development of normative reference values.

### 4.2 Applying MV perturbed the vestibular inputs and confirmed the sensory reweighting hypothesis

Applying either Uni or Bi on young and older adults significantly increased TR and PI, indicating a greater body sway during standing. This finding was regarded as a sign of temporary vestibular dysfunction, which was consistent with our published MV studies and previous studies involving galvanic vestibular stimulation (GVS) on young and older adults (Copeland et al., 2024; Lin et al., 2022; Lu et al., 2022; Woll et al., 2019; Zhang et al., 2024). Consequently, this study confirmed that the application of MV could perturb vestibular inputs and trigger temporary vestibular dysfunction, suggesting its potential value in simulating a vestibular‐disrupted environment in clinical evaluation. Additionally, applying MV increased SaEn_ML of older adults regardless of the SOT conditions they were in. These observations implied that the employment of MV not only increased body sway but also reduced its regularity during standing, resulting in poorer balance control than the baseline condition (Lin et al., 2022). According to the results of this study, the above‐described phenomenon was more pronounced among healthy older adults, indicating that ageing‐related vestibular deteriorations may cause them to be more sensitive to MV, as evidenced by greater alterations in balance control with the application of MV. Furthermore, our results confirmed the sensory reweighting hypothesis that vestibular perturbation altered the sensory weights allocated to each sensory channel (Chien et al., 2017; Peterka, 2002). While applying different types of MV during standing, vestibular inputs became unreliable; thus, the CNS automatically allocated more sensory weight to the proprioceptive system. Based on this hypothesis, the simultaneous visual and vestibular perturbation may further enhance the reliance on proprioceptive feedback, as proprioception became the sole reliable sensory input for maintaining balance when standing blindfolded with application of MV. However, it should also be noted that none of the participants took a step for balance or fell during data collection, indicating that proprioceptive inputs alone were sufficient for them to maintain balance despite simultaneous visual and vestibular perturbations. As a trade‐off, the CNS had to adjust the sensory contributions based on the availability of each sensory system (Assländer & Peterka, 2014). Two previous studies confirmed that instantaneous environmental changes led to the maladaptation of sensory reweighting, which resulted in postural instability while standing (Assländer & Peterka, 2016; Shindo et al., 2023). The present study first applied different types of MV to observe sensory reweighting dynamics and further supported the hypothesis that the process of sensory reweighting was accompanied by alterations in balance control, as reflected in both the magnitude and the regularity of CoG displacement.

Notably, an increase in TR under MV was only observed in the SOT‐2 condition, whereas the application of MV elevated PI and SaEn in both SOT‐1 and SOT‐2 conditions, suggesting a limitation in using TR as a measure for evaluating balance control. Compared with PI and SaEn, TR had the following drawbacks: (1) relatively low sensitivity: during unblindfolded standing, the effect of MV on TR did not reach the significance level, indicating that the measurement of TR might not be sensitive enough to detect subtle differences in balance control in an unblindfolded standing task (Tesio & Rota, 2019); and (2) relatively low comparability: in this study, TR measured the total distance of CoG displacement through a specific period for an overall evaluation of balance control. Since it was not normalized to the baseline value, the differences in TR across different MV conditions did not achieve statistical significance, but demonstrated corresponding tendencies (Panzer et al., 1995). Therefore, a complex standing task (blocked visual feedback) was necessary for the measurement of TR to highlight the effect of MV on balance control. Moreover, according to our results, there was no significant difference in TR between young and older adults, regardless of the conditions they stood in. In summary, the measurement of TR may not be an appropriate parameter to probe the effects of MV or ageing. In light of the above‐described findings, the measurements of PI and SaEn were more recommended to evaluate subtle differences in balance control.

### 4.3 Unilateral MV demonstrated a stronger impact on ML balance control than bilateral MV

According to our published studies that applied MV during standing or walking, Bi had a stronger effect on balance control than Uni in both young and older adults (Chien et al., 2017, 2016; Lin et al., 2022; Lu et al., 2022; Wang, Xie, et al., 2024). Interestingly, in this study, we found that the effect of MV on balance control in standing was direction‐dependent. Specifically, Uni demonstrated a greater impact on disturbing balance control in the ML direction than Bi, as evidenced by higher PI_ML and SaEn_ML under Uni, despite the non‐significant differences. In comparison to the baseline condition, applying Uni led to approximately 75% and 16% increases in PI_ML and SaEn_ML, respectively, in all participants, while applying Bi only increased PI_ML and SaEn_ML by 48% and 10%, respectively. Consequently, the above‐described findings suggested that the application of Uni resulted in an imbalance between bilateral vestibular feedback and disturbed balance control in the ML direction. This vestibular imbalance had been substantiated in unilateral vestibulopathy, which caused more vestibular‐related symptoms (e.g., vertigo and nystagmus) and losses of balance than bilateral vestibulopathy (Corre et al., 2024; Strupp & Magnusson, 2015). Moreover, Hatzilazaridis et al. (2019) also supported this hypothesis that the imbalance of vestibular feedback led to an asymmetry in the descending vestibulospinal drive to paraspinal muscles and generate a differential mechanical pull on the spine to develop scoliosis. In light of the above‐mentioned observations, it could be concluded that the postural stability in the frontal plane relied on symmetric inputs from bilateral vestibular organs (Xie et al., 2023). According to our previous research, we observed a relative insufficiency of the vestibular system, in which postural control deficits, particularly in the ML direction, might come from asymmetric vestibular inputs rather than the absence of vestibular feedback (Xie et al., 2025, 2024). In other words, relatively mild vertigo and imbalance experienced by patients with bilateral vestibulopathy may be attributed to the re‐establishment of an equilibrium of bilateral vestibular inputs (Takeda et al., 2024). Conversely, patients with bilateral vestibulopathy had a limited capability to re‐establish such equilibrium since there were considerable differences in vestibular inputs from both sides (Takeda et al., 2024). Therefore, it was reasonable to speculate that unilateral perturbation toward vestibular sensation (Uni) generated a pronounced postural instability in the frontal plane, even though the vestibular feedback on the contralateral side was intact (Xie et al., 2024). This phenomenon was also observed in the elderly with differential (asymmetric) vestibular deterioration (Sarvari et al., 2024). Our findings indicated that unilateral vestibular perturbation was superior in inducing vestibular imbalance and postural instability than bilateral vestibular stimulation, and could be used to simulate a sensory‐conflicted condition in future evaluation and rehabilitation (Lin et al., 2022; Xie et al., 2024).

### 4.4 Limitations

There were several limitations in the present study. One apparent limitation was the relatively small sample size. Nevertheless, based on the partial eta squared method, the effect sizes for all dependent variables were large in this study, suggesting the practical significance of our findings. In the near future, more participants will be recruited to fill this gap. The second limitation was that all participants in this study were healthy adults. Future studies should also include patient populations (e.g., vestibulopathy) to investigate whether the application of MV demonstrates a different impact on balance control compared to healthy individuals. Thirdly, it should be noted that vibrotactile stimulation at 100 Hz applied to mastoid processes may simultaneously manipulate both vestibular and proprioceptive feedback (Kavounoudias et al., 1999). Further studies should include the specific evaluation methods (e.g., video‐oculography) to distinguish between the manipulation of vestibular and proprioceptive inputs, and to ensure the successful vestibular stimulation by MV. Finally, in this study, there was no quantitative evaluation of potential ageing‐related sensorimotor deteriorations, which might also explain the alterations in balance control among older adults. Hence, in the future, detailed assessments of the sensorimotor system (e.g., electrophysiological and neurological methods) should be considered to quantify somatosensory and motor functions.

### 4.5 Conclusions

The present study revealed that different types of MV differently affected the balance control of healthy young and older adults. Notably, due to ageing‐related proprioceptive and vestibular deteriorations, older adults in a moderate/high level of physical activity still exhibited poorer balance control in standing, characterized by increased magnitude and irregularity of CoG displacement, compared to young adults. The application of MV perturbed vestibular inputs and induced sensory reweighting toward the proprioceptive system. Furthermore, Uni had a stronger impact on disturbing balance control in the frontal plane than Bi. Our findings could serve as fundamental evidence for the potential use of MV as an unpredictable vestibular perturbation tool in clinical settings.

## AUTHOR CONTRIBUTIONS

All data collections were performed at the Clinical Movement Analysis Laboratory at the University of Nebraska Medical Center. Haoyu Xie, Chuhuai Wang, and Jung Hung Chien completed the conception and design of this work. Haoyu Xie, Zhuo Wang, and Jung Hung Chien completed the data collection, analysis and interpretation of data for this work. Haoyu Xie and Jung Hung Chien acquired the funding to support this work. Haoyu Xie and Jung Hung Chien finished the original version of the manuscript. Zhuo Wang, Chuhuai Wang, and Jung Hung Chien revised the manuscript critically for important intellectual content. All authors have reviewed and approved the final manuscript, and agree to be accountable for all aspects of the work in ensuring that questions related to the accuracy or integrity of any part of the work are appropriately investigated and resolved. All persons designated as authors qualify for authorship, and all those who qualify for authorship are listed.

## CONFLICT OF INTEREST

None declared.

## Data Availability

The datasets generated during and/or analysed during the current study are available from the corresponding author on reasonable request.
